# Computational prediction of disordered binding regions

**DOI:** 10.1016/j.csbj.2023.02.018

**Published:** 2023-02-10

**Authors:** Sushmita Basu, Daisuke Kihara, Lukasz Kurgan

**Affiliations:** a Department of Computer Science, Virginia Commonwealth University, USA; b Department of Biological Sciences, Purdue University, West Lafayette, IN 47907, USA; c Department of Computer Science, Purdue University, West Lafayette, IN 47907, USA

**Keywords:** Intrinsic disorder, Disordered binding regions, Short linear motifs, Molecular recognition features, Protein-protein interactions, Protein-nucleic acids interactions, Protein-lipid interactions, IDP, intrinsically disordered protein, IDR, intrinsically disordered region, SLiM, short linear sequence motif, MoRF, molecular recognition fragment, CAID, Critical Assessment of Intrinsic Disorder, CASP, Critical Assessment of techniques for protein Structure Prediction, DL, deep learning, ML, machine learning, NN, neural network

## Abstract

One of the key features of intrinsically disordered regions (IDRs) is their ability to interact with a broad range of partner molecules. Multiple types of interacting IDRs were identified including molecular recognition fragments (MoRFs), short linear sequence motifs (SLiMs), and protein-, nucleic acids- and lipid-binding regions. Prediction of binding IDRs in protein sequences is gaining momentum in recent years. We survey 38 predictors of binding IDRs that target interactions with a diverse set of partners, such as peptides, proteins, RNA, DNA and lipids. We offer a historical perspective and highlight key events that fueled efforts to develop these methods. These tools rely on a diverse range of predictive architectures that include scoring functions, regular expressions, traditional and deep machine learning and meta-models. Recent efforts focus on the development of deep neural network-based architectures and extending coverage to RNA, DNA and lipid-binding IDRs. We analyze availability of these methods and show that providing implementations and webservers results in much higher rates of citations/use. We also make several recommendations to take advantage of modern deep network architectures, develop tools that bundle predictions of multiple and different types of binding IDRs, and work on algorithms that model structures of the resulting complexes.

## Graphical Abstract

**Figure:**
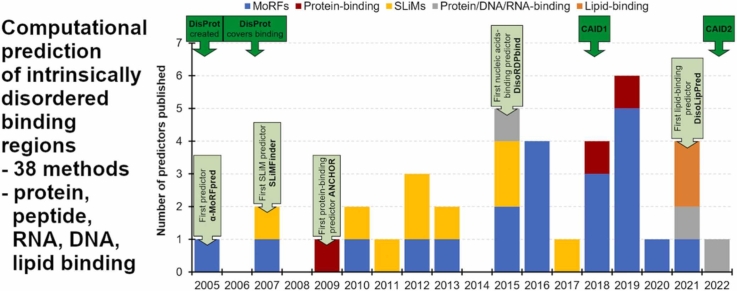

## 1 Introduction

Intrinsically disordered regions (IDRs) are segments in a protein sequence that lack stable structure under physiological conditions 1, 2, 3, 4. Intrinsically disordered proteins (IDPs) include one or more IDRs, and they could be fully disordered when an IDR covers the entire chain. IDPs are found across all domains of life, with a larger abundance in eukaryotic proteomes 5, 6, 7, 8. They play important roles in a plethora of cellular activities, complementing functions of the structured proteins and domains 9, 10, 11. Examples include cellular signaling and its regulation, translation, transcription, and phase separation 12, 13, 14, 15, 16, 17, 18, 19, 20, 21, 22. Being involved in key regulatory pathways, mis-regulation of IDPs and IDRs was shown to be associated with several human diseases 23, 24, 25, 26. Many of functions of IDPs involve interactions with a broad spectrum of partner molecules, including proteins, nucleic acids, lipids, metals, ions, carbohydrates and small-molecules 19, 21, 27, 28, 29, 30, 31. In that context, conformational plasticity of IDRs provides them with certain advantages compared to structured regions, such as ability of a single IDR to interact with multiple different partners, leading to an enrichment of IDPs among the hub proteins in the protein interaction networks 32, 33, 34, 35. Multiple types of interacting IDRs were categorized and characterized in the literature. Two of these types concern relative short sequences regions, molecular recognition fragments (MoRFs) and short linear sequence motifs (SLiMs). MoRFs are short IDRs that undergo disorder-to-order transition when interacting with proteins and peptides, i.e., they “morph” from disorder to order upon binding 36, 37, 38. Their length range varies across studies, with some works limiting their length to between 10 and 70 residues 37, 38, and other studies considering much shorter, 5–25 residues long, regions 36, 39. MoRFs are subdivided into multiple classes including α-MoRFs, β-MoRFs, γ-MoRFs and complex-MoRFs, based on the type of the secondary structure that they fold into upon binding, i.e., α -helix, β-sheet, irregular structures, and mixed secondary structures, respectively. SLiMs are relatively short sequence motifs represented by regular expressions that are found across multiple proteins 40, 41, 42. Majority of SLiMs are between 3 and 15 residues in length and many of them are disordered. They are associated with a variety of molecular interactions, primarily being involved in interactions with proteins and nucleic-acids [43]. Recent update of the ELM resource, a repository of eukaryotic linear motifs, reports over 3500 SLiMs that were curated from literature [40]. Moreover, human proteome was predicted to contain over 1 million binding motifs [44]. Another type of binding IDR called protean segments is defined by the IDEAL database [45]. These are short segments that are disordered in an unbound form and undergo folding upon binding with a partner molecule. The protean segments overlap with MoRFs and SLiMs but they are not limited in length like MoRFs, and do not have to be defined by regular expressions like SLiMs. The above three classes of binding IDRs are defined by their sequence features (length and motifs), modes of interactions with the partner molecule (coupled binding and folding), and binding to specific types of partners (proteins, peptides and nucleic acids). However, some interacting IDRs can be long, may not involve motifs, and may bind a variety of other molecules 30, 46. For instance, IDRs longer than 30 residues that bind proteins and peptides were classified as protein-binding IDRs [47].

While a huge number of binding IDRs occur in nature, only a relative handful of them has been annotated by biochemical experiments. More specifically, a few hundred IDRs with binding information are available in the DisProt database, the largest repository of functionally annotated IDRs [30]. Computational methods can help with closing this annotation gap. The limited collection of annotated binding IDRs can be used to develop and evaluate computational predictors, which then can be utilized to predict these regions for the millions of protein sequences that remain unannotated. This approach relies on the fact that the disordered nature of IDRs is intrinsic (i.e., encoded) in their underlying sequences 4, 48, 49, 50, making them predictable from the sequence. This has motivated development of numerous methods that accurately predict IDRs from the protein sequence 51, 52, 53, 54, 55, 56, 57, 58, 59, 60, 61, with over 100 methods that were developed to date [62]. Recent research has shifted from building disorder predictors to developing methods that predict binding IDRs. Similar to IDRs, recent study shows that binding IDRs also have compositional bias in their sequences [48], suggesting that they can be predicted directly from the sequence. Significance of these predictors is reflected by the inclusion of the assessment of the binding IDRs predictions in the recently completed community-organized Critical Assessment of Intrinsic disorder (CAID) experiment [63]. The CAID experiment evaluated 11 predictors of disordered binding regions; we discuss further details later.

Predictors of intrinsic disorder have been comprehensively surveyed and analyzed in a large number of studies 4, 64, 65, 66, 67, 68, 69, 70, 71, 72, 73, 74, 75, 76, 77. They were evaluated in a several comparative assessments, most notably as part of the community-driven efforts including the Critical Assessment of techniques for protein Structure Prediction (CASP) experiments 78, 79, 80, 81 and more recently the CAID experiment [63]. Disorder prediction was part of CASP between CASP5 in 2002 that evaluated six methods [81] and CASP10 in 2012 which assessed 28 predictors [78], compared to CAID that was performed in 2018 and compared 32 disorder predictors. In contrast, only a few reviews focus on prediction of binding IDRs while over three dozen of these methods were developed. A survey that covered 12 predictors of binding IDRs that were discussed together with over 30 disorder predictors was published in 2017 [82]. Two articles were published in 2019 83, 84. The first overviews 20 predictors of binding IDRs that target MoRFs, SLiMs, and other protein-binding IDRs, while omitting methods that target other types of interactions [83]. The second is a book chapter that describes 22 predictors of MoRFs, SLiMs, protein and nucleic acid binding regions, largely overlapping in scope with the other study [84]. We note that prediction of disordered binding region is gaining momentum in recent years, with 13 methods published since 2019. These factors motivate this systematic survey of predictors of binding IDRs. We provide a historical perspective, comprehensively enumerate current tools, categorize them based on architectures and their predictive targets, details predictive architectures for several tools that secured best results in the CAID experiment, highlight a few interesting observations concerning availability and impact of these tools, and offer several recommendations. Moreover, we fill the gap created after the surveys from 2019 and cover 38 methods that target a diverse set of ligands including peptides, proteins, RNA, DNA and lipids.

## 2 Historical overview

We perform an exhaustive literature search to identify a comprehensive collection of predictors of binding IDRs. We consider three main sources: i) extraction of methods that are covered in articles that focus on the prediction of binding IDRs and disorder functions 82, 83, 84, 124; ii) manual search of citations to these methods; and iii) manual search of the results produced by a relevant and broad PubMed search: (((Intrinsically disordered proteins) AND ((binding region) OR (binding residue)) AND (identification)), (((Intrinsic disorder) AND (binding region) AND (predictor)) OR ((MoRF) AND (prediction)), (((Intrinsic disorder) AND (short linear motif) AND (prediction)), ((Intrinsically disordered proteins) AND ((RNA binding) OR (DNA binding) OR (nucleotide binding))) AND ((binding region) OR (binding residue)) AND ((prediction) OR (identification)), ((Intrinsically disordered proteins) AND (lipid binding)) AND ((binding region) OR (binding residue)) AND ((prediction) OR (identification)). We combine results from these sources and remove duplicates, which results in a list of 38 methods that were published between 2005 to June 2022 (Table 1) 36, 39, 85, 86, 87, 88, 89, 90, 91, 92, 93, 94, 95, 96, 97, 98, 99, 100, 101, 102, 103, 104, 105, 106, 107, 108, 109, 110, 111, 112, 113, 114, 115, 116, 117, 118, 119, 120, 121, 122, 123. We first provide a historical overview of this area of research, which we follow by a discussion of several key aspects of these computational tools including their predictive models, popularity and availability.

**Table 1:** Predictors of binding IDRs grouped by types: MoRFs, protein-binding IDRs, SLiMs, protein/DNA/RNA-binding IDRs and lipid-binding IDRs. Methods are sorted chronologically in each type. 'Predictive architecture' column covers scoring functions (SF), regular expressions (Regex), hidden markov model (HMM)shallow machine learning (ML) and deep machine learning (DL). Specific ML and DL algorithms include neural network (NN), support vector machine (SVM), XGBoost, naive Bayes (NB), linear regression (LR), deep NN (dNN), long-short term memory network (LSTM) and multi-layer perceptron NN (mlpNN). 'Availability' covers webserver (WS), source code (SC), both (WS+SC) and never implemented (NA). 'URL' gives pages where a given method was available as of July 2022. 'Citations' includes total citations with annual citations inside brackets; these data were collected from Google Scholar in July 2022. For methods published in multiple articles, we use the reference with the highest citation count to avoid duplicate counting.

Target of prediction	Method name (year published)	Reference	Predictive architecture	Availability	URL	Citations total (per year)
MoRFs	α-MoRFpred (2005)	[39]	ML (NN)	NA	NA	647 (39)
	α-MoRFpred II (2007)	[85]	ML(NN)	NA	NA	317 (22)
	retro-MoRFs (2010)	[86]	SF (alignment)	NA	NA	43 (4)
	MoRFpred (2012)	87, 88	ML (SVM)	WS	http://biomine.cs.vcu.edu/servers/MoRFpred/	316 (32)
	MFSPSSMpred (2013)	[89]	ML (SVM)	WS+SC	The website does not work as of July 2022	55 (7)
	MoRF_CHiBi_ (2015)	[90]	ML (SVM)	WS+SC	https://gsponerlab.msl.ubc.ca/software/morf_chibi/	75 (11)
	DISOPRED3 (2015)	[91]	ML (SVM)	WS+SC	http://bioinf.cs.ucl.ac.uk/disopred	638 (92)
	MoRFC_HiBi_Web_ (2015)	[92]	ML (NB)	WS+SC	https://gsponerlab.msl.ubc.ca/software/morf_chibi/	35 (5)
	fMoRFpred (2016)	[36]	ML (SVM)	WS	http://biomine.cs.vcu.edu/servers/fMoRFpred/	112 (19)
	MoRF_CHiBi_ SYSTEM (2016)	[93]	ML (NB)	WS+SC	https://gsponerlab.msl.ubc.ca/software/morf_chibi/	93 (16)
	Predict-MoRFs (2016)	[94]	ML (SVM)	SC	https://github.com/roneshsharma/Predict-MoRFs	23 (4)
	Fang et al. (2018)	[95]	ML (SVM)	NA	NA	6 (2)
	MoRFPred-plus (2018)	[96]	ML (SVM)	SC	https://github.com/roneshsharma/MoRFpred-plus/wiki/MoRFpred-plus	41 (11)
	OPAL (2018)	[97]	ML (SVM)	WS+SC	http://www.alok-ai-lab.com/tools/opal/	50 (13)
	OPAL+ (2019)	[98]	ML (SVM)	WS+SC	http://www.alok-ai-lab.com/tools/opal_plus/	27 (9)
	en_DCNNMoRF (2019)	[99]	DL (dNN)	WS	The website does not work as of July 2022	9 (3)
	MoRF_MPM_ (2019)	[100**]**	ML (MPM)	SC	https://github.com/HHJHgithub/MoRFs_MPM	6 (2)
	MoRFPred_en (2019)	[101**]**	DL (dNN +dNN+SVM)	WS	The website does not work as of July 2022	5 (2)
	MoRF_MLP_ (2019)	[102**]**	DL (mlpNN + NB)	NA	NA	6 (2)
	SPOT-MoRF (2020)	[103**]**	DL (dNN)	WS+SC	http://sparks-lab.org/jack/server/SPOT-MoRF/index.php	24 (12)
	MoRF_CNN_ (2021)	[104**]**	DL (dNN)	NA	NA	0 (0)
Protein-binding	ANCHOR (2009)	105, 106	SF	WS+SC	http://anchor.enzim.hu	566 (44)
	ANCHOR2 (2018)	[107**]**	SF	WS+SC	http://iupred2a.elte.hu	754 (189)
	IDRBind (2019)	[108**]**	ML (XGBoost)	WS	https://idrbind.msl.ubc.ca/	1 (1)
SLiMs	SLiMFinder (2007)	[109**]**	Regex	WS	http://bioware.ucd.ie/∼compass/biowareweb/Server_pages/api.php	173 (12)
	SLiMProb (SLiMSearch 1.0) (2010)	[110**]**	Regex	WS	http://www.slimsuite.unsw.edu.au/servers/slimprob.php	10 (1)
	SLiMSearch 2.0 (2011)	[111**]**	Regex	WS	http://bioware.ucd.ie/∼compass/biowareweb/	77 (7)
	SLiMPred (2012)	[112**]**	ML (NN)	WS	http://bioware.ucd.ie/∼compass/biowareweb/	74 (8)
	SLiMPrints (2012)	[113**]**	SF (alignment)	WS	http://bioware.ucd.ie/∼compass/biowareweb/	84 (9)
	PepBindPred (2013)	[114**]**	ML (NN)	WS	The website does not work as of July 2022	31 (4)
	QSLiMFinder (2015)	[115**]**	Regex	WS	http://rest.slimsuite.unsw.edu.au/qslimfinder	17 (3)
	Song et al. (2015)	[116**]**	discriminative HMM	NA	NA	2 (1)
	SLiMSearch4.0 (2017)	[117**]**	Regex	WS	http://slim.icr.ac.uk/slimsearch/	68 (14)
Protein/DNA/RNA-binding	DisoRDPbind (2015)	118, 119	ML (LR)	WS	http://biomine.cs.vcu.edu/servers/DisoRDPbind/	118 (17)
	flDPnn (2021)	[120**]**	DL (dNN)	WS+SC	http://biomine.cs.vcu.edu/servers/flDPnn/	37 (37)
	DeepDISObind (2022)	[121**]**	DL (dNN)	WS	https://www.csuligroup.com/DeepDISOBind/	6 (6)
Lipid-binding	DisoLipPred (2021)	[122**]**	DL (dNN)	WS	http://biomine.cs.vcu.edu/servers/DisoLipPred/	6 (6)
	MemDis (2021)	[123**]**	DL (dNN)	WS+SC	http://memdis.ttk.hu/ https://github.com/brgenzim/MemDis	1 (1)

### 2.1 Historical progress in coverage of different types of interacting IDRs

**Fig. 1:**
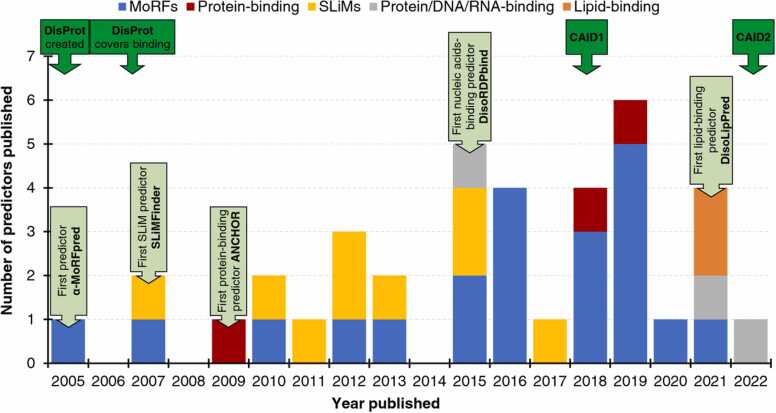
Timeline of the development of predictors of binding IDRs. Color-coded bars denote different prediction targets including MoRFs (blue), protein-binding regions (red), SLiMs (yellow), protein/DNA/RNA-binding regions (grey) and lipid-binding (orange) regions. Dark green callouts show major events that drive the development of these predictors. Light green callouts identify the first predictor for each ligand type.

We summarize historical overview in Fig. 1. The initial focus was primarily confined to the prediction of MoRFs and SLiMs, with 10 out of the 11 methods that were published before 2015 targeting these two types of binding IDRs. The very first method is α-MoRFpred that was developed by Keith Dunker’s group in 2005 [39]. It predicts α-helix-forming MoRFs by relying on the PONDR VL-XT-generated disorder predictions [58]. The main challenge at this point was lack of annotated MoRF regions, which had to be manually compiled from the data available in Protein Data Bank (PDB) 125, 126. The α-MoRFpred was developed using a small dataset of 14 MoRFs from 12 proteins, which were unlikely to represent a broader population of MoRF regions. An improved version of this algorithm, α-MoRFpred-II, was published two years later [85]. This predictor utilized a larger training dataset (102 MoRF regions from 99 proteins) and a machine learning algorithm, a shallow feed-forward neural network. However, implementation of the resulting predictor was not released, limiting its potential applications. The year 2012 marks the release of MoRFpred 87, 88, the first predictor that tackles prediction of generic MoRFs, irrespective of their type (as compared to α-MoRFs). This method has a more advanced design compared to the earlier tools. It uses a comprehensive sequence-derived input, which includes evolutionary profile and putative disorder, solvent accessibility and B-factors, that is processed by a support vector machine model. The model was trained on a large dataset of over 400 proteins with MoRF regions and the resulting predictor was released as a publicly accessible webserver, which is operational to this date.

Tools that extract/predict SLiMs were being developed in parallel to the efforts that target prediction of MoRFs. SLiMFinder, the first predictor of SLiMs, was published by Denis Shields’s lab in 2007 [109**]**. This method utilizes the SLiMBuild algorithm that constructs motifs, ranks them by their probability, and estimates their statistical significance. SLiMFinder offers options to restricts motif finding to specific regions of the protein sequence, such as IDRs that it predicts with the IUPred method [59], and is available in the form of a convenient webserver. Several other methods that produce SLiMs were developed subsequently, with majority of them including SLiMSearch 1.0 [110], SLiMSearch 2.0 [111**]**, SLiMPred [112**]**, SLiMPrints [113**]**, PepBindPred [114**]** and SLiMSearch 4.0 [117**]** developed by the labs of Denis Shields and Norman Davey.

With growing interest in prediction of binding IDRs, the focus has gradually shifted towards prediction of IDRs that interact with specific ligands, such as proteins, RNA, DNA, and lipids. ANCHOR, which was published by Zsuzsanna Dosztanyi’s lab in 2009, is the first method that predicts protein-binding IDRs 105, 106. ANCHOR is based on a scoring function that was derived by comparing disordered binding residues between their bound and unbound states. The prediction process is very fast and this method is available as a source code and a webserver. These factors undoubtedly contribute to high levels of popularity of this tool. DisoRDPbind, which was released by Lukasz Kurgan’s lab in 2015, is the first method that predicts nucleic acid binding IDRs 118, 119, 127. This tool relies on three relatively simple logistic regressions that are used to predict protein-binding, RNA-binding, and DNA-binding IDRs. The only other tools that target prediction of the nucleic acid-binding IDRs are flDPnn and DeepDISOBind that were released very recently 120, 121. They improve over the DisoRDPbind’s model by utilizing more sophisticated deep neural networks. The newest addition to the toolbox of predictors of binding IDRs are the two tools that predict lipid-binding IDRs, DisoLipPred [122**]** and MemDis [123**]**, which were released in 2021. Interestingly, they complement each other since MemDis focuses on IDRs in trans-membrane proteins while DisoLipPred predicts lipid-binding IDRs that specifically exclude trans-membrane regions. Lastly, we note that there are no predictors for the protean regions.

### 2.2 Major events

The timeline in Fig. 1 can be divided into two distinct periods, a first-generation period before 2015 and a second-generation period that started in 2015. The first-generation period is characterized by a relatively slower pace of the development efforts, with on average 1.1 new methods published per year, and focus on a small subset of the binding IDR types, such as MoRFs and SLiMs. The efforts intensified in the second-generation period, with on average 3.4 methods published per year and a broader coverage of binding IDR types, which include MoRFs, SLiMs, protein-binding, nucleic acid-binding, and lipid-binding IDRs. This increase results from an improved availability of ground-truth annotations of binding IDRs. The early methods, such as α-MoRFpred, α-MoRFpred-II, MoRFpred, and MoRF_CHiBi_, primarily relied on parsing data from PDB, which is rather difficult since it requires processing atomic-level data, aggregation at residue level and comparing across multiple structures given that PDB files are redundant and often cover fragments of protein sequences. Moreover, these data are also limited since PDB centers on providing access to structured proteins and regions. The first database of disordered proteins, DisProt, was established in 2005 128, 129. It started with a few hundred IDPs that were annotated based on published experimental data. It took several years before the annotations of binding were added and a sufficiently large number of these annotations was collected. By early 2010 s the amount of the accumulated binding IDRs was sufficient to develop and test predictive tools, and the second-generation tools, such as DisoRDPbind, flDPnn, DeepDISObind, DisoLipPred, and MemDis, rely on DisProt to source training and test datasets. These annotations are easier to collect compared to PDB data since they are reported at the residue level and mapped into full protein sequences. Moreover, they are more diverse, allowing to collect data to develop methods for more types of binding IDRs.

Besides the development and growth of DisProt, the other significant event that stimulates efforts to develop predictors of binding IDRs is the CAID experiment, which was held in 2018 and included evaluation of the these predictors [63]. CAID is the first community-driven evaluation of accuracy of predictions of binding IDRs, which suggests growing interest in this area. Several best-performing methods secured area under the ROC curve (AUC) values> 0.7, including ANCHOR2 [107**]** with AUC = 0.742, DisoRDPbind’s model for the protein-binding IDRs [118**]** with AUC = 0.729, MoRF_CHiBi_Light_ [93] with AUC = 0.720, and MoRF_CHiBi_Web_ [92] with AUC = 0.702. Overall, among the 11 methods which participated in the CAID’s binding IDR prediction assessment, five perform above a baseline level: ANCHOR2, DisoRDPbind, the two versions of MoRF_CHiBi_, and OPAL [97]. We refrain from reporting predictive performance of individual methods based on their respective publications since these results should not be directly compared due to differences in the datasets, metrics and test procedures used. We also note several drawbacks of CAID. It performs evaluation of binding predictions in a ligand agnostic way, i.e., different types of binding IDRs were clumped together. We note that the five above-baseline methods target prediction of protein-binding IDRs, benefitting from the fact that 72% of the binding annotations in the CAID dataset are protein-binding. Overall, this challenge shows substantial potential for future improvements. Interestingly, some of these limitations are being addressed in the currently pending CAID2 experiment (https://idpcentral.org/caid). CAID2 expands the assessment of predictions of binding IDRs by introducing assessment of ligand-specific prediction that cover protein-binding and nucleic-acid binding. This will likely result in a further growth in the efforts to generate more diverse and more accurate methods.

## 3 Predictors of disordered binding regions

Table 1 covers several important aspects of the 38 predictors of binding IDRs, such as their predictive architectures, modes of availability, and popularity quantified with citations. We categorize these methods into five groups based on the target of their predictions: MoRFs, SLiMs, protein-binding regions, lipid binding regions, and protein/DNA/RNA-binding regions. The methods in the latter category identify three types of binding IDRs, those that interact with proteins, with DNA, and with RNA.

### 3.1 Predictors of MoRFs

The largest group of predictors of binding IDRs focuses on the MoRF regions, with 21 out of the 38 methods (55%) in this category (Table 1). The defining feature of MoRFs is their ability to transition to structured conformation upon binding to proteins and peptides, which implies that the underlying interaction-dependent structure differentiates them from other binding IDRs. While the first MoRF predictor targeted α-MoRFs, majority of the subsequent tools were designed to target all types of MoRFs, irrespective of how they fold upon binding.

The most popular (i.e., based on annual number of citations listed in Table 1) and available to the end users MoRF predictors include MoRFpred [87], MoRF_CHiBi_ [90], DISOPRED3 [91], fMoRFpred [36] and OPAL [97]. We briefly summarize MoRFpred in Section 2.1. MoRF_CHiBi_ was first published in 2015 and has been successively improved by the same authors 90, 92, 93, ultimately resulting in the MoRF_CHiBi_ SYSTEM that is composed of three predictors: MoRF_CHiBi_, MoRF_CHiBi_Light_ and MoRF_CHiBi_Web_ [93]. MoRF_CHiBi_Light_ and MoRF_CHiBi_Web_ rely on predictions from MoRF_CHiBi_, but MoRF_CHiBi_Light_ does not utilize computationally expensive PSSM profiles, which makes it much faster than MoRF_CHiBi_Web_. Thus, users of the MoRF_CHiBi_ SYSTEM have an option to apply a fast MoRF_CHiBi_Light_ version or slower and more accurate MoRF_CHiBi_Web_ version.

DISOPRED3 is a popular predictor of disorder that includes an option to predict MoRF regions [91]. The disorder predictor uses a small neural network to combine SVM-based DISOPRED2 model [130**]**, neural network specialized to predict long IDRs, and a nearest neighbor-based classifier that takes advantage of similarity to annotations in a training dataset. DISOPRED3 applies a separate SVM-based model that uses information extracted from the input sequence and its PSSM profile to predict MoRFs.

Another popular MoRFs predictor is OPAL [97]. This is a meta-predictor that averages results produced by two MoRF predictors: MoRF_CHiBi_ and a relatively slow PROMIS [97]. The fMoRFpred tool represent an opposite approach, with a simpler architecture and fast runtime [36]. This method utilizes a basic SVM-based model that relies on fast-to-compute putative disorder predicted with IUPred [131**]** and putative secondary structure generated with the fast single-sequence version of PSIPRED [132**]**.

### 3.2 Predictors of SLiMs

Majority of predictors that target SLiMs rely on regular expressions to identify these motifs in protein sequences. This is the second most populous category of predictors, with 9 methods published to date (Table 1). The most popular and available to the end users SLiMs predictors include SLiMFinder [109**]**, which we described in Section 2.1, and SLiMSearch 4.0 [117**]**. The latter tool is a successor of the SLiMSearch 1.0 [110**]** and SLiMSearch 2.0 [111**]** methods. SLiMSearch 4.0 is an advanced framework that identifies SLiMs using likelihood-based scoring of motifs, sequence conservation, functional enrichment analysis using Gene Ontology (GO) terms, and filters that consider putative disorder generated with IUPred, surface accessibility when structure is available, and overlap with Pfam domains [117**]**. Moreover, SLiMSearch 4.0 identifies SLiMs in a taxonomy-aware manner, focusing on around 70 model species that include human, yeast, mouse, fruit fly, *C. elegans*, and *A. thaliana*. We also note a recently released SLiMSuite package [133**]**, which provides convenient access to multiple tools for discovery and characterization of SLiMs: SLiMProb [110**]**(also known as SLiMSearch 1.0), SLiMFinder [109**]** and QSLiMFinder [115**]**. Besides these regular expression-based tools, there are two methods that utilize machine-learning models to predict SLiMs: SLiMPred [112**]** and PepBindPred [114**]**. Both methods apply bidirectional recurrent neural network models and rely on information extracted from sequence-derived predictions of secondary structure, intrinsic disorder and solvent accessibility. PepBindPred additionally performs docking between the interacting molecules.

### 3.3 Predictors of protein, RNA, DNA and lipid-binding regions

There are three predictors which target protein-binding IDRs: ANCHOR 105, 106, which we discussed in Section 2.1, ANCHOR2 [107**]** and IDRBind [108**]**. The two ANCHOR methods are arguably the most popular predictors of binding IDRs. ANCHOR2 improves over ANCHOR by extending its scoring function with additional terms, which results in a more accurate model.

Recent years observed the push to develop methods that predict IDRs that interact with nucleic acids and lipids. There are three tools that predict DNA/RNA/protein-binding IDRs: DisoRDPbind 118, 119, flDPnn [120], and DeepDisoBind [121**]**, and two tools that predict lipid-binding IDRs: DisoLipPred [122**]** and MemDis [123**]**. These methods, with the exception of DisoRDPbind, apply state-of-the-art deep learning models that we explore in Section 3.4.

### 3.4 Predictive architectures

We identify five categories of predictive architectures that are used to implement predictors of binding IDRs: scoring functions (SF), regular expressions (regex), shallow machine learning (ML) algorithms, deep-learning (DL) algorithms and meta-predictors; see “predictive architecture” column in Table 1. These categories are in line with similar analyses for the disorder predictors 67, 71, 72, 74.

The SF-based models use pre-defined functions to combine evolutionary and biochemical features that are estimated from protein sequences. Key characteristics of these functions are that they utilize relatively few parameters and rely on explicit formulas that are typically derived from biophysical principles underlying interactions. Examples include retro-MoRF [86] and SLiMPrints [113**]** that utilize scoring functions based on the conservation extracted from multiple sequence alignments, and ANCHOR and ANCHOR2 that use interaction energy-based features 105, 107.

Regex-based models are exclusively used for the prediction of SLiMs 109, 110, 111, 115, 117. Regex is a sequential combination of symbols and characters that represents a pattern for a short string that can be efficiently searched in a longer string (i.e., amino acid sequence). Using regex, prediction of SLiMs boils down to search for short motifs in a given protein sequence, followed by ranking to find statistically significant hits, and filtering to identify motifs in a specific part of the sequence, e.g., disordered region. Prominent examples of the regex-based predictors include SLiMFinder [109**]** and SLiMSearch 4.0 [117].

ML and DL, the two most numerous categories, utilize machine learning algorithms to generate predictive models from training datasets. There are 28 of them in total including 9 DL models and 19 ML models. These algorithms depend on the quality and size of the training datasets since they utilize the ground truth from these datasets to optimize predictive models, such that they minimize differences between predictions and the corresponding grounds truth. Shallow ML algorithm are the traditional classifiers that in general produce smaller models and require less training data than the deep learning algorithms. Over a half of the shallow ML methods (i.e., 10 out of 19) utilize models produced with the support vector machine (SVM) algorithm 36, 87, 89, 90, 91, 94, 95, 96, 97, 98. Other algorithms include linear regression 118, 119, naïve Bayes 92, 93, XGBoost [108**]**, minimax probability machine [100**]**, and shallow neural networks 39, 85, 112, 114. The DL algorithms are neural networks with topologies that include multiple/many hidden layers and which also typically use more sophisticated types of neurons and utilize modern types of architectures, such as convolutional and recurrent networks. These models usually involve a large number of parameters (i.e., weights associated with the connections between neurons in the network) and thus they need large datasets to properly train these parameters. The DL-based predictors of binding IDRs apply a variety of architectures including convolutional 99, 101, 104, 121, bidirectional recurrent [122**]**, recurrent Long Short-Term Memory (LSTM) [103**]**, hybrid of convolutional and recurrent LSTM [123**]**, as well as deep fully-connected perceptron network 102, 120. We note that methods developed since 2020 exclusively utilize the DL models. Part of the reason why these models could be developed is that a sufficient amount of training data has become available in recent years, driven mostly by the substantial growth of the DisProt database. When a sufficient amount of training data became available and given the breakthroughs in the designs of deep network architectures in the past decade and the resulting high-levels of their predictive performance, unsurprisingly, researchers in this field have shifted to adopt DL algorithms instead of traditional ML. This is likely also motivated by the recent influx and success of DL-based predictors of intrinsic disorder. Notably, the top-performing disorder predictors in CAID [134**]** include flDPnn [51], SPOT-Disorder2 [52], rawMSA [53] and AUCpred [54], all of which rely on the DL models. Furthermore, recent empirical study finds that the DL models in general produce more accurate disorder predictions when compared to the shallow ML models [64], which provides a strong justification to develop these models for prediction of binding IDRs.

Finally, there are several meta-predictors which are defined as methods that combine predictions of binding IDRs produced by multiple predictors. The underlying objective is to provide more accurate results when compared to the results produced by the input predictors. This approach was used to develop several popular and accurate disorder predictors 135, 136, 137, 138, 139, 140, 141. We identify four meta-predictors, all of which predict MoRFs, including MoRF_CHiBi_Web_, MoRF_CHiBi_ SYSTEM, OPAL and OPAL+ 90, 93, 97, 98. The focus on MoRFs can be explained by the fact that the most and large number of predictors target this category of binding IDRs, providing a deep pool of input predictions for the meta-method.

Lastly, we detail predictive models of the five methods that performed well in the CAID experiment [63]: ANCHOR2, DisoRDPbind, two versions of MoRF_CHiBi_ method, and OPAL. ANCHOR2 [107**]** is the SF-based model that improves over its predecessor, ANCHOR 105, 106. ANCHOR implements SF that quantifies differences in basic biophysical properties of disorder binding residues between their bound and unbound state. It combines the putative disorder information generated by IUPred with estimates of pairwise interaction energy of disordered residues with globular proteins and local disordered sequence segments. ANCHOR2 uses a computationally efficient linear function to combine the interaction energy estimation from ANCHOR with two new terms that estimate energy for interaction with binding surface of globular proteins and presence of a disordered sequence. This results in a more accurate model that still retains the small computational footprint of ANCHOR.

DisoRDPbind [118**]** is a shallow ML method that utilizes three logistic regression models to predict RNA-binding, DNA-binding and protein-binding propensities, one regression for each ligand type. These regressions use a common input profile generated from the sequence that includes information about hydrophobicity and net charge, putative disorder produced with IUPred [59], putative secondary structure generated by a single-sequence version of PSIPRED [142**]**, and sequence complexity computed by the SEG algorithm [143**]**. This profile is processed to generate inputs for the regressions using sliding-windows with sizes that are optimized for specific ligand types.

MoRF_CHiBi_ SYSTEM [93] is also a shallow ML predictor but it features a multi-layer architecture. The bottom layer implements the base MoRF_CHiBi_ model that uses a Bayes rule to combine MoRF predictions from two SVM models, one that is trained directly on sequences and the other that relies on similarities between sequences. The second layer implements the MoRF_CHiBi_Light_ prediction [93] by using a Bayesian model to fuse predictions from the base MoRF_CHiBi_ with the predictions of disorder from ESpritz-DisProt [55]. The third layer implements the MoRF_CHiBi_Web_ prediction [93] that again uses a Bayesian model to combine the base MoRF_CHiBi_, the ESpritz-DisProt predictions and the conservation derived from the sequence using PSI-BLAST [144**]**. Benchmarking done by the authors suggests that MoRF_CHiBi_Light_ produces more accurate predictions than the base MoRF_CHiBi_, while MoRF_CHiBi_Web_ further increases accuracy but at substantially higher computational cost due to the calculation of the conservation [93].

OPAL [97] is a meta-predictor that averages results produced by the base MoRF_CHiBi_ model and PROMIS, a relatively slow MoRF predictor developed by the authors of OPAL. PROMIS predicts MoRFs using an SVM model based on putative solvent accessibility, secondary structure and torsional angles predicted from the input sequence with SPIDER2 [145**]** and a PSSM profile generated from the sequence with PSI-BLAST. The need to compute the PSSM profiles results in a relatively long runtime.

We highlight the fact that these models are rather diverse. They utilize a variety of predictive architectures and different inputs that are derived from the sequence. They also vary in terms of their runtime. The CAID experiment reports that ANCHOR2 and DisoRDPbind take around 1 second to predict one protein, MoRF_CHiBi_Light_ takes a few seconds, and the other two methods require two order of magnitude more runtime due to the use of PSI-BLAST, i.e., about 100 seconds for MoRF_CHiBi_Web_ and over 500 seconds for OPAL [63].

### 3.5 Availability and impact

Availability of these predictors to a broad scientific user-group is an important factor to facilitate research on binding IDRs. Table 1 provides details on implementations and whether they are currently available, i.e., as of July 2022 when we collected these data. There are two types of implementations: webserver (WS) and source code (SC). WS is available online via a web browser or programmable interface, typically does not require installation of any software, and performs all computations on the server side. While webservers are usually accessed via webpages, in a few cases (e.g., SLiMPred, SLiMPrints and QSLiMFinder) the access is based on the representational state transfer (REST) interface. SC have to be downloaded, installed/compiled and run on user’s hardware. While WSs are easier to use, they are typically limited to prediction of a single or a few proteins at the time and could be difficult to embed into other bioinformatics platforms if they lack programmable interface. On the other hand, SC usually can be setup to perform predictions on a larger scale and is easier to incorporate into other bioinformatics software, but it can be challenging to install and requires hardware to run. We collect the location of these WS and SC resources as per information given in the respective publications and check their availability. We find 27 methods that have working WS and/or SC implementations. Among them 13 methods are available solely as WS, 3 as SC and 11 as both WS and SC. There are 4 methods which were once functional but as of July 2022 did not work, and 7 methods that were never implemented for public use. The corresponding 71% rate of availability (27 out of 38 methods) is relatively high, higher than the 65% availability rate for disorder predictors [62], and much higher than the approximately 40% rate for other related predictors of protein-binding and nucleic acid binding residues 146, 147.

We analyze impact/use of these methods, which we quantify in using citations collected from Google Scholar as of July 2022 (Table 1). We provide total number of citations as well as an annual count, where the latter is a better metric to compare impact/use of different methods. The predictors published from 2020 onwards are too new to reliably measure their citation data, hence, we exclude them from the below analysis. We find that methods which offer WS and/or SC implementation are cited much more often (median annual citations = 12), compared to methods which were never made available (median annual citations = 3). Moreover, among the methods which are currently functional, the tools that provide both WS and SC are cited more (median annual citations = 16) compared to the methods that provide only WS (median annual citations = 12) and only SC (median annual citations = 4). The higher popularity of predictors implemented as WSs is because they are arguably more convenient for majority of users who have limited computational resources and are less computer savvy to be able to install and run software locally. Methods with no implementations suffer low citations, revealing that availability directly influences the level of use and impact. These observations suggest that future methods should be made available as both WS and SC to maximize impact. Moreover, we find that among the methods which have/had WS and/or SC implementations, the ones which are currently non-functional receive median annual citations of 4, which is 4 times lower than the functional methods. This means that it is vitally important to maintain availability after methods are released.

We briefly discuss impact/use of individual tools. Predictor of protein-binding IDRs, ANCHOR2, is the most highly cited method, both in terms of annual citations (189) and total citations (754). We note that ANCHOR2′s publication also introduces a popular disorder predictor, IUPred2, which likely inflates the above number; this is also why we use median annual citations to compare groups of tools. There are 9 predictors that were cited over 100 times and 4 of them were cited over 500 times. These observations should be considered with a pinch of salt, since these tools were published in 2016 or earlier and had more time to accumulate citations when compared to newer methods. However, this reveals a significant amount of interest in using these methods.

## 4 Summary and outlook

IDRs interact with many different molecular partners including proteins, DNA, RNA, lipids, small molecules, carbohydrates, and metals. The knowledge of these interactions is rather limited, which motivates development of computations tools that predicts them from the readily available protein sequences. This comprehensive survey of sequence-based predictors of binding IDRs covers a wide range of interacting partners. We identify and summarize a large collection of 38 predictors that consider 5 different types of interacting IDRs. The MoRF predictors are the largest category with 21 methods, followed by 9 SLiM predictors, 3 predictors of protein-binding IDRs, 3 methods that predict protein/DNA/RNA binding IDRs and 2 predictors of lipid-binding IDRs. We find that these methods rely on a diverse range of predictive architectures that include scoring functions, regular expressions, machine learning models and meta-predictors, where about three-quarters of them utilize machine learning algorithms. We observe a couple of recent trends to develop deep network-based models and to extend coverage to new types of interacting IDRs, such as RNA, DNA and lipid binding regions. We also note a high rate of availability of these methods, with over 70% that are provided to the end users as either webservers and/or standalone code. Furthermore, we analyze relation between availability and impact/use of these methods. We find that methods which are more broadly available, as both webserver and source code, are substantially more cited/used when compared to those that are available in either format, while methods that do not offer a publicly available implementation suffer low use/citations. Moreover, we also find that the availability should be maintained since tools that were originally made available and are currently not functional observe a large drop in the use/citations. The latter observations strongly suggest that future predictors should be made available in both formats upon publication and should be maintained after publication.

While IDPs interact with a broad range of molecular partners, we show that the current predictors are largely focused on two types of binding IDRs, MoRFs and SLiMs. A particularly acute situation concerns prediction of nucleic acid and lipid-binding IDRs, where only a handful of methods are available. The prediction of small molecules-, carbohydrates-, and metal-binding IDRs is not feasible at the moment, given a very small amount of ground truth data. The need to develop new predictors of DNA and RNA binding regions is further motivated by the inclusion of this prediction category in the pending CAID2 experiment. Consequently, one of the key future directions would be to diversify the development efforts to more uniformly cover different types of binding IDRs.

Results of the recently completed CAID assessment show that predictors of binding IDRs offer modest levels of predictive performance [63], suggesting that there is a large room for improvement. We observe that none of the methods that participated in this evaluation use deep learning models. The recent influx of the deep learning-based predictors of binding IDRs will likely result in improved predictive quality. This claim stems from a recent study that empirically demonstrates that deep learning-based predictors of intrinsic disorder significantly outperform other types of models [64]. The drive to use deep learning models is also motivated by the growing and successful use of these models in related areas of bioinformatics [148**]**, such as prediction of protein-protein interactions 149, 150, 151 and protein function 152, 153, 154. We envision that majority of future predictors of binding IDRs will likely rely on deep neural networks. We encourage the developers to consider modern network topologies, such as the recently developed transformers [155**]**, that were used to very accurately predict protein structures [156**]**.

Some IDPs include IDRs that interact with different types of ligands and yet most of the current methods cover a single ligand type. Consequently, users are forced to use multiple methods and convert between different output formats to obtain a complete prediction. These difficulties could be alleviated with solutions that bundle multiple predictors, however, the only such solution to date is the DEPICTER webserver [157**]**. Moreover, there are only a handful of methods that predict IDRs that bind to multiple ligand types, such as DisoRDPbind, flDPnn and DeepDISObind, that target protein, RNA and DNA-binding IDRs. Consequently, we advocate for the development of new tools that address predictions of multiple and many different types of binding IDRs. Furthermore, some IDRs can bind multiple partner types, which corresponds to multi-label (multi-output) learning. Prediction of such multifunctional IDRs is possible with the DMRpred method, although this tool does not provide types of binding partners [158**]**. Thus, new tools that would cast this prediction as multi-labels problem should be developed. We note that multi-labels predictors are widely used in related areas, such as prediction of subcellular localization 159, 160, 161, 162, nucleic acid binding proteins [163**]**, enzymatic functions [164**]**, and ion channel types [165**]**.

Prediction of the binding IDRs in protein sequences should be followed by modelling structures of the corresponding complexes (i.e., IDRs fold upon binding). While computational protein docking has been extensively pursued over the past several years [166**]**, studies that investigate docking with IDPs are lagging behind since IDPs are difficult to model. Daisuke Kihara’s lab developed a pioneering approach for IDP-protein docking, IDP-LZerD 167, 168. This method produces a docking model from the 3D structure of the receptor and the sequence of interacting IDP. Docking an IDP is conceptually similar to protein-small peptide docking, but technically more challenging because conformation of the IDP on the receptor’s surface has to be predicted. In IDP-LZerD, this is done by docking and stitching short protein fragments taken from the binding IDR. Moreover, a recent benchmark study that evaluates three methods capable of docking with IDPs, IDP-LZerD 167, 168, CABS-Dock [169**]** and AlphaFold-Multimer [170**]**, shows that they accurately identify location of the binding site but struggle with atomic-levels details of the structure [171**]**, suggesting that further research is needed.

Lastly, databases like D^2^P^2^ [172**]**, MobiDB 173, 174, 175, 176 and DescribePROT [177**]** provide convenient access to pre-computed predictions of disorder for millions of proteins. However, they typically contain a limited number of binding IDR predictions, with DescribePROT covering the most diverse range that includes putative protein, RNA and DNA-binding IDRs. This coverage should be extended in the future as more methods that cover a broader range of binding IDRs will be developed. In turn, this effort motivates the development of runtime-efficient predictors that can be used to perform predictions on such large scale. Examples of current fast tools include ANCHOR2, DisoRDPbind and fMoRFpred, that were shown to produce predictions in about 1 second per protein in the CAID experiment [63].

## Funding

LK was funded in part by the 10.13039/100000001National Science Foundation (DBI2146027 and IIS2125218) and the Robert J. Mattauch Endowment funds. DK acknowledges supports from the 10.13039/100000002National Institutes of Health (R01GM133840 and 3R01GM133840–02S1) and the 10.13039/100000001National Science Foundation (CMMI1825941, MCB1925643, DBI2146026, IIS2211598, DMS2151678, and DBI2003635).

## CRediT authorship contribution statement

**Sushmita Basu**: Data curation; Formal analysis; Investigation; Methodology; Validation; Writing – original draft. **Lukasz Kurgan**: Conceptualization; Data curation; Formal analysis; Funding acquisition; Investigation; Project administration; Supervision; Validation; Writing – original draft; Writing – review & editing. **Daisuke Kihara**: Conceptualization; Funding acquisition; Investigation; Supervision; Writing – original draft; Writing – review & editing.

## Conflicts of interest

The authors declare no conflicts of interest.
